# Detecting Sulfuric and Nitric Acid Rain Stresses on *Quercus glauca* through Hyperspectral Responses

**DOI:** 10.3390/s18030830

**Published:** 2018-03-09

**Authors:** Shanqian Wang, Xiuying Zhang, Yuandan Ma, Xinhui Li, Min Cheng, Xiaomin Zhang, Lei Liu

**Affiliations:** 1 Jiangsu Provincial Key Laboratory of Geographic Information Science and Technology, International Institute for Earth System Science, Nanjing University, Nanjing 210023, China; shanqianwang@163.com (S.W.); xinhui___li@163.com (X.L.); chengmin007008@163.com (M.C.); zxm08ygy@163.com (X.Z.); liulei_nju_geo@163.com (L.L.); 2 The Nurturing Station for the State Key Laboratory of Subtropical Silviculture, Zhejiang Agriculture and Forestry University, Hangzhou 311300, China; mayuandan@gmail.com; 3 Jiangsu Center for Collaborative Innovation in Geographical Information Resource Development and Application, Nanjing 210023, China

**Keywords:** acid rain stress, green peak area index (GPAI), damages, pH levels, leaf hyperspectral data

## Abstract

Acid rain, which has become one of the most severe global environmental issues, is detrimental to plant growth. However, effective methods for monitoring plant responses to acid rain stress are currently lacking. The hyperspectral technique provides a cost-effective and nondestructive way to diagnose acid rain stresses. Taking a widely distributed species (*Quercus glauca*) in Southern China as an example, this study aims to monitor the hyperspectral responses of *Q. glauca* to simulated sulfuric acid rain (SAR) and nitric acid rain (NAR). A total of 15 periods of leaf hyperspectral data under four pH levels of SAR and NAR were obtained during the experiment. The results showed that hyperspectral information could be used to distinguish plant responses under acid rain stress. An index (green peak area index, GPAI) was proposed to indicate acid rain stresses, based on the significantly variations in the region of 500–660 nm. Light acid rain (pH 4.5 SAR and NAR) promoted *Q. glauca* growth relative to the control groups (pH 5.6 SAR and NAR); moderate acid rain (pH 3.0 SAR) firstly promoted and then inhibited plant growth, while pH 3.0 NAR showed mild inhibitory effects during the experiment; and heavy acid rain (pH 2.0) significantly inhibited plant growth. Compared with NAR, SAR induced more serious damages to *Q. glauca*. These results could help monitor acid rain stress on plants on a regional scale using remote sensing techniques.

## 1. Introduction

Acid rain, or acid deposition, has become one of the most serious environmental problems around the world. Along with Europe and North America, China has become the third region severely polluted by acid rain. Sulfur dioxide (SO_2_) and nitrogen oxides (NO_X_), mainly emitted from the combustion of fossil fuel and traffic emissions, are the precursors of acid rain [1,2]. Through complex photochemical reactions, these two pollutants can be converted to sulfate (SO_4_^2−^) and nitrate (NO_3_^−^) in precipitation [3]. In Europe and North America, acid rain is dominated by nitric type because of the huge oil consumption by automobiles, while sulfuric acid rain dominates in China due to the use of coal as the main energy source [4]. However, in recent years acid rain in China has gradually transformed from sulfuric type to the mixed sulfuric and nitric type, and the areas affected by acid rain have extended from the south to the north with the rapid economic growth and industrialization [5,6]. Since sulfate and nitrate have different effects on plants, the actual acid composition of the precipitation should be fully considered when studying plant responses to acid rain.

Forests are greatly influenced by acid rain pollution. Acid rain can directly exert deleterious effects on plant phenotype and physiological characteristics and then inhibit the normal growth of plants [7,8,9]. Moreover, it can indirectly influence plant growth through acidifying surface waters and soils [7,10]. The biological effects of acid rain on plants are numerous and complex, including visible symptoms of injury and invisible effects [11]. The plant population structures and community functions are affected by altering the activities of antioxidant enzyme and the contents of free amino acids [12,13], affecting leaf nutrient balance and influencing seedling emergence and growth [14,15]. Furthermore, acid rain can change the amount of leaf surface wax and its chemical composition [16], destroy membrane permeability [5], and influence chlorophyll concentration and dark respiration rate [17,18].

Hyperspectral technology which is rapid, non-destructive, repetitive and relatively inexpensive, compared with biochemical analyses, provides an approach to measure plant optical properties [19]. With the development of hyperspectral technology, the spectral resolution of hyperspectral sensors is sufficient to detect subtle changes in the spectral behavior of various biochemicals [20]. The physiological status of plants can directly affect to both leaf structures and leaf pigments, so the changes of physiological behavior of plants induced by stress will result in the differences in hyperspectral reflectance [21,22,23], which provides the theoretical basis for monitoring the health status of plants using hyperspectral information [24,25]. 

Spectral reflectance has been widely used to detect plant stresses due to its potential of identifying different plant conditions [26,27]. For example, Ranjan et al. assessed the effects of plant nitrogen stress on wheat through hyperspectral indices, and found that the spectral reflectance of leaves with different nitrogen content varied greatly in the 400–700 nm range [28]; Zhang et al. investigated plant (wheat, cabbage, locust and ash) responses under heavy metal (Cu) stress, and showed that the reflectance in the 530–570 nm and 800–900 nm regions displayed significant changes [29]; Delalieux et al. found that biotic stress (*Venturia inaequalis* exposure) made the spectral reflectance of apple trees show significant changes in the regions of 580–660 nm and 685–715 nm [30], and Di Vittorio and Biging found that the reflectance of pine needles damaged by ozone (O_3_) significantly changed in visible region, particularly for the 535–635 nm and 670–685 nm ranges [31]. 

Hyperspectral technology has been proven to be an effective way to monitor acid rain stress on plants. Xie et al. indicated that the spectral difference of *Elaeocarpus glabripetalus Merr* leaves under acid rain could be identified using the narrow band spectrum [32]. Cheng et al. found the first derivative spectrum of *Schima superba* under acid rain treatment showed a ‘blue shift’ (a red edge shift toward shorter wavelengths, where the red edge is the point at which the maximum spectral reflectance slope occurs in the spectrum) [33]. Song et al. discovered that *Masson pine* showed a ‘red shift’ (a red edge shift toward longer wavelengths) and the spectral reflectance at green peak (550 nm) and red edge region (690–750 nm) decreased with the increasing intensity of acid rain [34].

These results suggest that different plants subjected to acid rain showed dissimilar spectral responses, but detailed studies on long-term responses of plants under different acid rain acidity stresses are lacking. In addition, the studies mentioned above focused on sulfuric acid rain (SAR) stress on plants, and there are few studies at present on the responses of plants to nitric acid rain (NAR) stress. Furthermore, although many vegetation indexes have been proposed to monitor the stresses, few indexes were designed to monitor acid rain stress on vegetation. For instance, the physiological reflectance index (PRI) was first formulated for characterizing the carotenoids and light use efficiency [35]. Moreover, normalized difference vegetation index (NDVI) is not a good indicator of stress since it is only accurate for leaf area index (LAI), biomass and chlorophyll determination at relatively low level, while it shows a saturation effect at higher levels of those factors [36,37]. Therefore, it is urgent to propose a new spectral index to indicate the acid stresses on plants.

Taking *Quercus glauca*, a typical subtropical tree species in southern China, as the subject of the controlled experiment, this study aims to: (i) seek the hyperspectral bands that are sensitive to acid rain and propose an indicator of acid rain stress, (ii) detect and compare the long-term responses of *Q. glauca* to sulfuric and nitric acid rain stresses at a leaf scale, and (iii) provide a theoretical basis and scientific references for monitoring plant responses under acid rain pollution on a regional scale.

## 2. Materials and Methods

### 2.1. Experiment Design

The experiment was conducted in a greenhouse located in Lin’an District, Zhejiang Province, China (30°14′ N, 119°42′ E) and was arranged as a completely randomized design. One-year seedlings of *Q. glauca* were purchased from a local sapling company, and a total of 80 *Q. glauca* seedlings were selected and transplanted into plastic flowerpots in early May, 2013. The height and the upper and lower diameters of the flowerpot are 30 cm, 30 cm and 27 cm, respectively. The soil in flowerpots is the typical red soil of Lin’an District in Zhejiang Province. These seedlings were randomly separated into two equal groups, and then respectively sprayed with two types of acid rain: sulfuric acid rain (SAR, SO_4_^2−^:NO_3_^−^ = 8:1) and nitric acid rain (NAR, SO_4_^2−^:NO_3_^−^ = 1:8). Each type of acid rain had four pH level treatments: pH 5.6 (Control Group), pH 4.5, pH 3.0 and pH 2.0. Every treatment had 10 pots of seedlings as replicates. To avoid contamination from the neighboring acid rains, each treatment had a 0.5 m spacing. The greenhouse was not enclosed to maintain the same temperature as the outside (Table 1). No artificial lights were used in the greenhouse, and all the seedlings were illuminated by natural light. The average monthly rainfall in Lin’an District from 1951 to 2008 was used to determine the amount of sprayed acid rain (Table 1). From 1 June 2013, seedlings were sprayed with acid rain once a week until 22 July 2014.

### 2.2. Spectral Measurement

The leaf hyperspectral reflectance data (350–2500 nm) were measured using a FieldSpec FR hand-held spectroradiometer (Analytical Spectral Devices, Inc. (ASD); Boulder, CO, USA) equipped with a special fibre optic probe for plant leaves (the leaf probe has a built-in tungsten quartz halogen lamp which provides a stable light source). The sampling interval of this device is 1.4 nm with a resolution of 3 nm for the region of 350–1000 nm, and 2 nm with spectral resolution of 10–12 nm for the region of 1000–2500 nm. The spectral data were then interpolated by ASD ViewSpecPro software for post-processing to produce values at each nanometer interval, and then the interpolated data were exported in txt format.

Hyperspectral data were measured approximately every 20 days, and in total 15 periods of data were obtained over the experiment. We measured five of the 10 seedlings of each pH level treatment at each measurement period, and the remaining five seedlings were used as spares. Seven leaves per seedling were evenly selected for a quick spectral measurement on a workbench with a black background. A 99.9% reflectance white standard panel was used to obtain a reference signal every ten minutes to determine the relative reflectance of each leaf. During spectral measurement, the fibre optic probe should be vertical downward and slightly contact the leaf. Each leaf was measured 10 times, and the spectral reflectance of each leaf sample was determined to be the average of the 10 spectral measurements.

### 2.3. Sensitive Bands Detection 

The abnormal data deviating from the mean more than three times of the standard deviation are removed. For each measurement period, the average spectral reflectance of each pH level (350 spectra per pH level) of SAR and NAR is calculated respectively. To compare differences in spectral reflectance between pH 5.6 treatment and other three pH level treatments, one-way analysis of variance (ANOVA) is used to detect the hyperspectral bands which were sensitive to acid rain. For each type of acid rain, 60 hyperspectral spectra (there are 15 measurement periods, and 1 average spectrum per pH level at each measurement period) of four pH levels are tested by ANOVA using SPSS software, version 19.0 (IBM, Armonk, NY, USA), and the analysis is performed in the 350–1000 nm region, which has relatively high resolution and less noise. Dunnett’s test for ANOVA is used to calculate *p*-values to determine significant differences. Spectral bands with significant difference (*p* < 0.05) between different treatments may indicate the spectral responses of *Q. glauca* seedlings to acid rain stress and would be selected as the sensitive bands.

### 2.4. Hyperspectral Index Construction 

From the studies of Ranjan et al. [28], Zhang et al. [29], and Delalieux et al. [30], the sensitive bands are not unique and are usually continuous wavelengths. Using a single band to study the long-term effects of acid rain on *Q. glauca* will not make full use of the obtained sensitive bands information. Furthermore, the accuracy and stability of indicator will reduce. In line with this assumption, an area index (AI) is proposed in this study to characterize the influence of acid rain stress on *Q. glauca* according to the sensitive bands resulted from Dunnett’s test. The integral between the spectral curve and *X*-axis in a region of continuous sensitive bands is calculated, and the AI is defined as Equation (1):

(1) $$\mathrm{AI}=\int_{a}^{b}R\left(\lambda \right)d\lambda ,$$

where *λ* is wavelength, *R*(*λ*) is the spectral reflectance at *λ*, *a* and *b* are the starting and stopping points of the selected sensitive bands.

As two classical vegetation indices, normalized difference vegetation index (NDVI) [38], and physiological reflectance index (PRI) [35] have been used to identify plant stresses induced by other factors, such as water, salinity, heavy metal and drought [39,40,41,42]. To validate the applicability of AI for acid rain stress, NDVI, PRI and AI at different pH levels are compared against each other using an ANOVA followed by the least-significant difference (LSD) test to quantify their sensitivities to acidity at the early stage (24 July 2013), medium stage (1 January 2014) and late stage (22 July 2014) of the experiment. The index with significant difference (*p* < 0.05) between different pH treatments is considered sensitive to acid rain. NDVI and PRI are defined as Equations (2) and (3):

(2) $$\mathrm{NDVI}=\frac{R_{800}\;-\;R_{670}}{R_{800}\;+\;R_{670}},$$

(3) $$\mathrm{PRI}=\frac{R_{531}\;-\;R_{570}}{R_{531\;}+\;R_{570}},$$

where *R*_800_, *R*_670_, *R*_531_ and *R*_570_ are the spectral reflectance at 800 nm, 670 nm, 531 nm and 570 nm, respectively.

## 3. Results

### 3.1. Sensitive Hyperspectral Bands Detection and Hyperspectral Index Construction 

**Figure 1:**
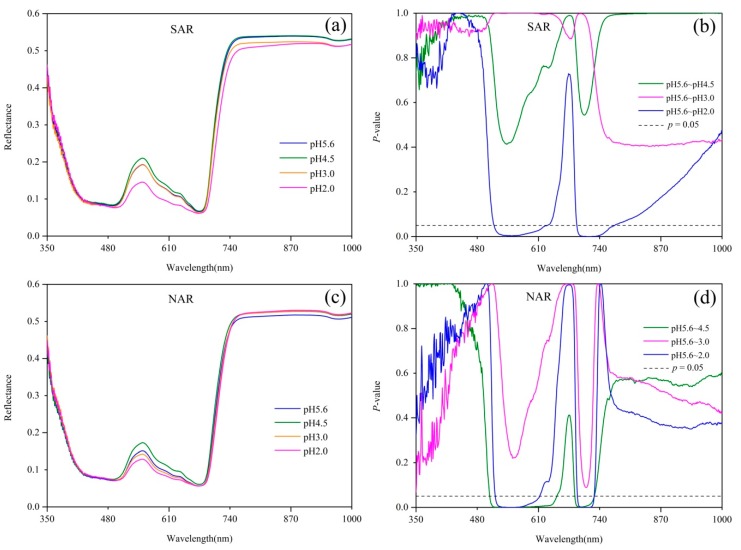
(**a**) Spectral reflectance and (**b**) its difference of *Q. glauca* under 4 pH levels of SAR, and (**c**) Spectral reflectance and (**d**) its difference of *Q. glauca* under 4 pH levels of NAR. *p* = 0.05 line indicates statistically significant difference between treatments according to Dunnett’s test results.

Spectral reflectance and the results of Dunnett’s test are shown in Figure 1. The reflectance of *Q. glauca* under acid rain stresses in both visible region and the near-infrared (NIR) region varied significantly, particularly near the green peak (550 nm). Compared with the control group (pH 5.6 acid rain treatment), the reflectance of *Q. glauca* under pH 4.5 and pH 2.0 acid rains had much more difference than that of pH 3.0. The reflectance of pH 4.5 treatment near the green peak was higher but pH 2.0 was lower than pH 5.6, while pH 3.0 did not show significant difference from pH 5.6 (Figure 1a,c). Compared with pH 5.6 treatment, the reflectance within 514–637 nm and 692–771 nm had significant differences (*p* < 0.05) under pH 2.0 SAR treatment, but no spectral bands showed significant differences under pH 4.5 and pH 3.0 SAR treatments (Figure 1b). Compared with pH 5.6 NAR stress, pH 4.5 treatment had significant differences (*p* < 0.05) in the range of 505–656 nm and 688–728 nm, while pH 3.0 had no significant bands, and pH 2.0 were in 517–621 nm and 693–728 nm (Figure 1d).

From the sensitive bands analyzed above, the reflectance in green region and red edge region were significantly impacted by acid rain stresses. Particularly, the reflectance of *Q. glauca* near green peak had an obvious and regular response to acid rain stress. The reflectance around green peak showed a trend of increasing first and then decreasing as the acidity increased. Considering the sensitive bands resulting from Dunnett’s test (Figure 1) and the regular changes of plant spectra in the green region, a complete reflectance peak in the spectral curve was used to calculate the area index. The area index covers the region of 500–660 nm, thus it is called green peak area index (GPAI). The GPAI is calculated according to Equation (1), where *a* = 500 and *b* = 660.

**Figure 2:**
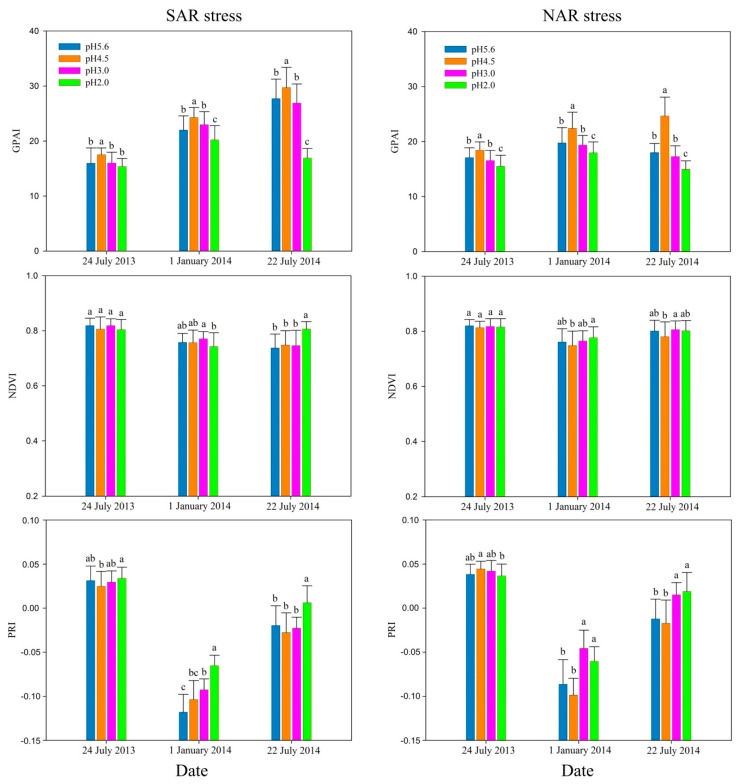
The difference of green peak area index (GPAI), normalized difference vegetation index (NDVI) and physiological reflectance index (PRI). Different letters above the columns indicates statistically significant difference (*p* < 0.05) among treatments according to LSD test results.

Figure 2 shows that in the early stage (24 July 2013) of the experiment, the indexes have poor sensitivity to acidity, but among the three indexes, GPAI showed the highest sensitivity to acidity for both SAR and NAR stresses. With the strengthening acidity, GPAI changed obviously. It showed significant difference (*p* < 0.05) between pH 5.6 and pH 4.5, pH 4.5 and pH 3.0 treatments under SAR stress, and between pH 5.6 and pH 4.5, pH 4.5 and pH 3.0, pH 3.0 and pH 2.0 treatments under NAR stress. NDVI between different pH level treatments did not show significant difference (*p* < 0.05) for both SAR and NAR stresses, while PRI showed significant difference (*p* < 0.05) only between pH 4.5 and pH 2.0 treatments, but had no significant difference (*p* < 0.05) between adjacent pH level treatments.

All the three indexes were more sensitive to acidity in the medium stage (1 January 2014) of the experiment than the early stage. GPAI showed significant differences (*p* < 0.05) between all adjacent pH level treatments. NDVI was significantly different (*p* < 0.05) between pH 3.0 and pH 2.0 treatments under SAR stress, and between pH 4.5 and pH 2.0 treatments for NAR stress. PRI had significant differences (*p* < 0.05) between the adjacent pH level treatments of pH 3.0 and pH 2.0 for SAR stress, and pH 4.5 and pH 3.0 for NAR stress.

In the late stage (22 July 2014) of the experiment, GPAI was still the most sensitive to acidity with significant differences (*p* < 0.05) between all adjacent pH level treatments. Among adjacent pH level treatments, NDVI was only significantly different (*p* < 0.05) between pH 3.0 and pH 2.0 treatments for SAR stress, and pH 4.5 and pH 3.0 treatments for NAR stress, while PRI obtained the similar results as NDVI.

GPAI showed significant changes with decreasing pH, and it had significant differences (*p* < 0.05) between all the adjacent pH level treatments except for pH 3.0 and pH 2.0 SAR on 24 July 2013. Among the adjacent pH level treatments, NDVI only had significant differences (*p* < 0.05) between pH 3.0 and pH 2.0 on 1 January 2014 and 22 July 2014 for SAR stress, and pH 4.5 and pH 3.0 on 22 July 2014 for NAR stress. PRI was only significantly different (*p* < 0.05) between pH 3.0 and pH 2.0 on 1 January 2014 and 22 July 2014 for SAR stress, and pH 4.5 and pH 3.0 on 1 January 2014 and 22 July 2014 for NAR stress. GPAI increased firstly and then decreased with acidity increase for both SAR and NAR stresses in all the three stages, while NDVI did not show an obvious regular and PRI overall presents an increasing trend except on the 24 July 2013 for NAR stress.

### 3.2. Indicator of GPAI on the Responses of Q. glauca under SAR Treatment

**Figure 3:**
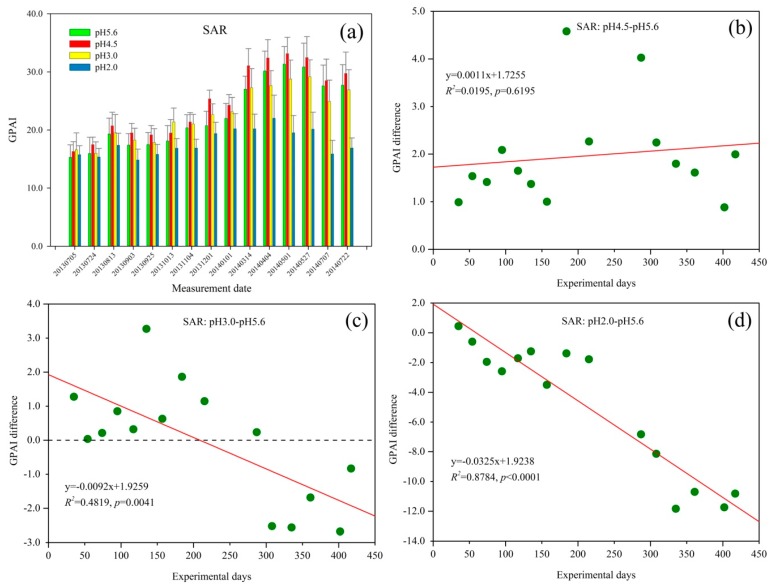
(**a**) Green peak area index (GPAI) trend of *Q. glauca* under sulfuric acid rain (SAR) treatment; and (**b**–**d**) the GPAI difference between different stress levels of SAR.

The GPAI of *Q. glauca* under SAR treatment is shown in Figure 3a. Overall, the GPAI of *Q. glauca* under all SAR treatments increased over time until 4 April 2014. It then decreased slightly but increased again at the end. Figure 3a shows that GPAI varied seasonally with relatively high values in August 2013 and May 2014. Among the four pH levels, the GPAI of pH 4.5 SAR treatment was the highest while pH 2.0 was the lowest.

The GPAI of *Q. glauca* under pH 4.5 SAR treatments was higher than that of pH 5.6 throughout the experiment (Figure 3b). The GPAI difference showed distinct seasonal characteristics, which increased slightly in the initial few measurement periods followed by a decreasing trend, and then increased again. With the spraying time extending under pH 4.5 SAR, the GPAI difference started to decrease at about 290 days of acid rain treatment (13 March 2014) but began to increase after July 2014.

Compared with the control group, *Q. glauca* under pH 3.0 SAR stress had higher GPAI at first (Figure 3c). The GPAI difference between pH 3.0 and pH5.6 also varied seasonally, decreasing at first and then increasing at about 75 days of treatment, until the eighth measurement period (1 December 2013). Subsequently, the GPAI difference decreased to a negative value at about 300 days of acid rain treatment (April 2014), and reached to −2.0 with mild increasing trend at the end.

As Figure 3d shows, the GPAI difference of pH 2.0 SAR only was positive at the first measurement. With the extension of the acid rain spraying time, the GPAI difference rapidly decreased and reached almost −12 at the end of this experiment. Although the stress of pH 2.0 SAR on *Q. glauca* had been increasing during the experiment, GPAI difference was also characterized by obvious seasonal variations. After decreasing at the initial stage of the experiment, the GPAI difference of *Q. glauca* under pH 2.0 SAR slightly increased at about 120 days of spraying acid rain. The GPAI difference then dropped rapidly and eventually stabilized around a value.

### 3.3. Indicator of GPAI on the Responses of Q. glauca under NAR Treatment

**Figure 4:**
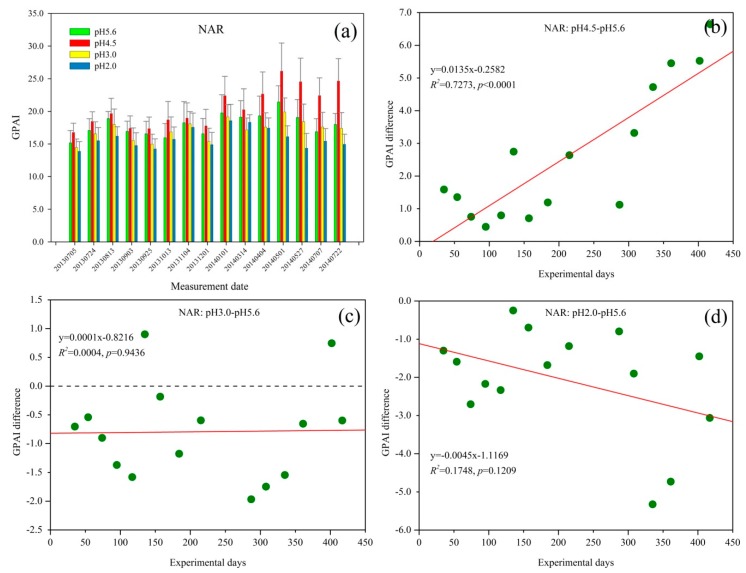
(**a**) Green peak area index (GPAI) trend of *Q. glauca* under nitric acid rain (NAR) treatment; and (**b**–**d**) the GPAI difference between different stress levels of NAR.

The GPAI of *Q. glauca* under NAR treatments increased with fluctuation over time, and then slightly decreased from 1 May 2014 (Figure 4a). However, with increasing acid rain spraying time, GPAI decreased from 1 May 2014 until 22 July 2014. Similar to SAR treatment (Figure 3a), the GPAI also has a seasonal trend with relatively high values in August 2013 and May 2014, and the *Q. glauca* under pH 4.5 NAR have the highest GPAI values while the pH 2.0 NAR treatment have the lowest.

GPAI difference between pH 4.5 and pH 5.6 NAR was positive and exhibited an increasing trend during the experiment (Figure 4b). This suggested that pH 4.5 NAR could promote *Q. glauca* growth and the promoting effect increased with the spraying acid rain time when contrasted to the control group. Similar to pH 4.5 SAR stress (Figure 3b), the GPAI difference of *Q. glauca* treated with pH 4.5 NAR also varied with the seasons.

Figure 4c shows that the GPAI difference between pH 3.0 and pH 5.6 NAR was negative at the initial stage of the experiment, with obvious seasonal fluctuations. However, it did not significantly decrease during the experiment. This suggested that pH 3.0 NAR inhibited *Q. glauca* at first relative to the control group, and the inhibitory effect was at a low level throughout the experiment. In addition, it can be found from Figure 4b,c that there was a tolerant pH threshold between pH 4.5 and pH 3.0 NAR for *Q. glauca*.

For *Q. glauca* under pH 2.0 NAR condition, GPAI difference was obvious negative at the initial time of the experiment and showed decreasing trend with the increasing treatment time (Figure 4d). Similarly, the GPAI difference between pH 2.0 NAR and pH 5.6 NAR also exhibited seasonal fluctuations. The GPAI difference was higher than that in Figure 3d at first few measurement periods, which suggested that the damage of pH 2.0 NAR to *Q. glauca* was more severe than pH 3.0 NAR.

## 4. Discussion

### 4.1. The Influence of Acid Rain on Spectral Reflectance and the Indicator of GPAI 

Acid rain can significantly influence the spectral reflectance of *Q. glauca*, especially in the yellow edge region, blue edge region and green region (Figure 1), which was consistent with the conclusions of previous studies [43,44]. These spectral fields are closely related to chlorophyll, carotenes and xanthophyll pigment absorption [43,45]. Compared with the control group (pH 5.6), the influence of pH 3.0 acid rain was not significant, and pH 2.0 acid rain caused a significant decrease in the green region, while the reflectance of pH 4.5 treatments increased. Plant’s reflectance is closely related to its chemical composition, morphology and structure. Heavy acid rain can leach K^+^, Ca^2+^ and Mg^2+^ from plant leaves, and then affect the synthesis of chlorophyll [46]. Additionally, H^+^ in acid rain can change the cellular pH balance, cause direct damage to leaf tissue, and then affect the normal function of plant cells, such as photosynthesis and respiration [16]. Once acid rain reaches the ground, it may cause the acidification of water and soil, and then affect plant growth [10]. For light acid rain, the positive effects of S and N on plant growth are more significant than the negative effect of H^+^, which make the seedlings treated with pH 4.5 acid rain have higher reflectance in green region relative to the control group, while the comprehensive effect of moderate acid rain was not significant [47,48].

Most plants with healthy green leaves have high reflectance in the green range (500–600 nm) of the light spectrum [25]. The spectral signature of plant could be affected by the physiological status [21,49], and the reflectance at green peak (550 nm), yellow edge (550–582 nm) and blue edge (450–530 nm) will decrease when plants health is inhibited [24,50]. This experiment showed the leaves with visible damage, such as the necrotic, and the yellowing and wilting leaves, had lower reflectance near green peak (550 nm) and therefore lower GPAI relative to healthy leaves, which was consistent with previous studies. For example, Naidu et al. investigated the leaf spectral reflectance changes between virus (*Grapevine leafroll-associated virus-3*) infected grape plants and the uninfected plants, and found that the reflectance of infected leaves with symptoms had a significant decrease in the green peak (near 550 nm) compared with healthy leaves (uninfected) [51]. Du et al. found leaf reflectance of *Cupressus funebris* and *Cunninghamia lanceolata* near the green peak (550 nm) decreased gradually from the young leaves to the old leaves and the dead leaves [52]. From our experiment and previous studies, the lower GPAI indicate the poorer health status of *Q. glauca*, and the positive values of GPAI difference between experimental groups and control group (pH 5.6) suggest certain levels of acid rain have positive effects on *Q. glauca* relative to the control group.

From the index difference between the adjacent pH level treatments (Figure 2), GPAI has more significant changes with increasing acidity, compared to NDVI and PRI. NDVI is significantly correlated with chlorophyll content and leaf area index, and usually the lower NDVI is associated with poorer health of plants [53]. As an indicator of photosynthetic efficiency, PRI is sensitive to the xanthophyll cycle, and an increasing PRI with an increasing photosynthetic efficiency [35,54]. Among four pH level treatments, GPAI of pH 2.0 treatment is the lowest on 22 July 2014. However, NDVI and PRI were the highest under pH 2.0, which is contrary to the fact that *Q. glauca* under pH 2.0 suffered the most significant damage with large area of withered leaves at the end of the experiment. These results indicated that GPAI was the most sensitive to acidity among the three indexes in the early stage, medium stage and late stage of the experiment, and it can be more accurate to track acid rain stresses on *Q. glauca* than NDVI and PRI.

### 4.2. Responses of GPAI to SAR and NAR Stresses 

GPAI of *Q. glauca* under SAR and NAR treatments increased over time at all pH levels until 4 April 2014 and 1 May 2014, respectively (Figure 3a and Figure 4a). This may be because the initial stage of this experiment was the plants’ growing season and plants need more nutrients of N and S than the other periods. At this stage, the element of S and N in SAR and NAR acted as fertilizers, thus they had a positive effect on *Q. glauca* [55,56,57]. Acid rain can destroy the wax and cuticle of plant leaves, which can cause physiological metabolism disorder and even death of plants [17,58,59]. After several months of acid rain treatment, the resistance of *Q. glauca* to bad environment and the synthesis ability of chlorophyll seems to have weakened, so the damage caused by acid rain to plants is relatively severe in spring [47]. Consequently, the GPAI began to decrease in April 2014, especially the GPAI of pH 2.0 treatment. With the fertilizer requirements for plant growth increased, the GPAI of *Q. glauca* showed an increasing trend after July 2014. The suitable temperature and relative humidity in May and August could improve enzyme activity of plants and then affect the growth of plants, which resulted in the relatively high values of GPAI in August 2013 and May 2014 (Figure 3a and Figure 4a) [47]. It is worth noting that, the GPAI of *Q. glauca* under SAR stress varied more greatly than NAR, suggesting *Q. glauca* was more sensitive to SAR than NAR. In addition, the GPAI of seedlings under SAR treatment was higher than that of NAR treatment at almost all stress levels, because smaller amount of sulfur fertilizer have more significantly influence on plant relative to nitrogen fertilizer [60]. Sulfur deficiency will reduce the utilization rate of nitrogen fertilizer for plants [61,62]. Therefore, for *Q. glauca* seedlings treated with NAR, nitrogen fertilizer did not benefit the plants well due to the sulfur deficiency.

### 4.3. GPAI as an Indicator for Assessing the Stress of SAR and NAR on Q. glauca at pH 4.5 Level 

The influences of pH 4.5 SAR and NAR on *Q. glauca* were positive due to the S and N nutrition for plants [55,60], thus GPAI difference between pH 4.5 treatments and control group showed an increasing trend over time. Usually, the plant’s morphological changes lag behind the physiological responses when it is subjected to environmental stresses, so the effect of acid rain on *Q. glauca* are observed in reflectance some time later after spraying acid rain [47]. Consequently, the seasonal variations of GPAI difference lagged behind the GPAI in Figure 3a and Figure 4a, and relatively high values of GPAI appeared in October 2013 and July 2014. At the beginning of experiment, *Q. glauca* showed certain responses to acid rain with a mild decrease of GPAI difference. Meanwhile, plant adaptive mechanisms against acid rain were triggered to protect plant from the further damage [58]. Furthermore, acid rain had fertilization effect on *Q. glauca*, therefore the promoting effect of acid rain was stronger than its inhibition and the GPAI difference increased subsequently. When the fertilizer demand of *Q. glauca* reduced, the GPAI difference started to decrease in March 2014. Additionally, the low temperature and the damage from acid rain declined chlorophyll synthesis efficiency, and then resulted in the decrease of GPAI difference [47]. The GPAI difference began to rise again after July 2014 as the demand for fertilizer increased [63].

In comparison with pH 4.5 SAR treatment, the GPAI difference under pH 4.5 NAR did not increase but slightly decreased in the initial several measurement periods. This may be due to the fertilization effect of S made the promotion of pH 4.5 SAR be stronger than its inhibition. However, sulfur deficiency resulted in the low utilization rate of nitrogen fertilizer, so *Q. glauca* under pH 4.5 NAR did not absorb N well [61]. Furthermore, H^+^ in NAR had negative effect on *Q. glauca* [5]. Figure 4b showed that the GPAI difference increased more quickly with a higher value (7.0) at the end and a higher slope (0.0135) of the fitted curve than that in Figure 3b (value = 2.0; slope = 0.0011). However, the GPAI of seedlings under NAR were lower than that of SAR at pH 4.5 level. This suggested that pH 4.5 NAR had more obviously positive effects on *Q. glauca* than pH 4.5 SAR relative to respective control groups, but the seedlings treated with SAR had better growth than those under NAR stress at pH 4.5 level. On the bases of the nitrogen and sulfur fertilizer offered by pH 5.6 NAR and pH 5.6 SAR, the increase of N (pH 4.5 NAR) may have more significant effects on *Q. glauca* than S increase (pH 4.5 SAR) [60]. However, on the whole, the influence of sulfur deficiency on plants were greater than nitrogen deficiency since nitrogen absorption could be influenced by sulfur [61,62].

### 4.4. GPAI as an Indicator for Assessing the Stress of SAR and NAR on Q. glauca at pH 3.0 Level 

Under pH 3.0 SAR, GPAI difference decreased from positive to negative values with seasonal fluctuations (Figure 3c). The fertilization effects of acid rain were predominant at the beginning. Acid rain could damage wax and cuticle of leaves, leach the Ca^2+^, K^+^, and Mg^2+^ in the leaves while Mg^2+^ is an essential element for the synthesis of chlorophyll, so long-term acid rain stress would cause injury to leaf tissue and its functions, and then inhibit the growth of *Q. glauca* [17,64,65]. Unlike the effect of pH 3.0 SAR, although pH 3.0 NAR inhibited *Q. glauca* at the beginning of the experiment, it did not obviously enhance over time (Figure 4c). Although *Q. glauca* suffered the same degree of damage from H^+^ in SAR and NAR at pH 3.0 level, pH 3.0 NAR had a negative effect on seedlings at first. This may be due to the utilization rate of nitrogen fertilizer was affected by sulfur deficiency, and thus the fertilization effect of pH 3.0 NAR was diminished [61].

GPAI difference in Figure 3c and Figure 4c decreased at first due to the damage from H^+^, and then resumed after adjustment, mainly due to the adaptive mechanisms of plants. The osmotic adjustment substance of soluble sugar and proline would increase to mitigate the damage when plants suffering from environmental stresses [58,66]. Since pH 3.0 acid rain was more harmful than pH 4.5 acid rain, the GPAI difference under pH 3.0 acid rain decreased earlier after increase than that of pH 4.5 acid rain (Figure 3b,c and Figure 4b,c) [55]. With the increase of acid rain spraying time, the direct damage of acid rain and the injury caused by soil acidification changed the influence of pH 3.0 SAR on *Q. glauca* from promotion to inhibition in April 2014 [47,67].

### 4.5. GPAI as an Indicator for Assessing the Stress of SAR and NAR on Q. glauca at pH 2.0 Level 

Under pH 2.0 acid rain stress, GPAI difference significantly decreased with the increasing experimental days. Heavy acid rain (pH 2.0 SAR and pH 2.0 NAR) had obviously inhibited the growth of *Q. glauca* throughout the experiment, and the inhibitory effect significantly enhanced over time. GPAI difference became negative value just at about 50 experimental days, which was much earlier than pH 3.0 SAR treatment. This is because heavy acid rain could cause damage or necrosis of plant tissues at the beginning of this experiment, and the plants did not recover due to the continuous spraying of acid rain and soil acidification [16,68,69]. Furthermore, heavy acid rain could restrain the synthesis of chlorophyll by leaching Mg^2+^ in leaves, destroy or disintegrate mitochondria in the leaf cells and uncouple oxidative phosphorylation, and then slow down the photosynthetic rate, increase the dark respiration rate and transpiration rate of plants, resulting in the excessive depletion of organic matter in plants and even death from starvation [55,70,71,72]. Compared with *Q. glauca* under pH 3.0 acid rain, GPAI difference between pH 2.0 and pH 5.6 treatments increased later after decreasing in the initial stage, which may be due to seedlings under pH 2.0 acid rain needed a longer time for adjustment (Figure 3c,d and Figure 4c,d).

With the experimental days increasing, pH 2.0 NAR rapidly declined GPAI difference (Figure 4d, slope = −0.0045). GPAI difference in Figure 3d (slope = −0.0325) decreased more quickly and was stable around −12.0 at the end of the experiment, while the GPAI difference in Figure 4d reached to −3.0 and still had a large fluctuation. As seen from this experiment, *Q. glauca* obviously responded to heavy acid rain at the initial stage due to the strong acidity, and *Q. glauca* may suffer heavy and even irreversible damage with the treatment time increasing [5,63]. Some studies showed that under severe acid rain stress, destructive superoxide anion radicals (O_2_^−^) were produced in plants, which unbalanced the coordinated effect of the antioxidant enzyme system, and ultimately increased the permeability of the plasma membrane [3,5]. This could change the cellular pH balance, drop the activities of membrane protective enzymes and the content of membrane protective substances [5]. Consequently, the structure of chloroplasts and mitochondria are destroyed, leading to the leakage of intracellular electrolytes, the disorder of vegetable metabolism and the inhibition of nutrient synthesis [5,73].

At the end of this experiment, *Q. glauca* under pH 4.5 SAR and pH 4.5 NAR treatments grew well, with luxuriant foliage, while the leaves under pH 3.0 SAR and pH 3.0 NAR were partially wilted and yellowed. Some of the *Q. glauca* sprayed with pH 2.0 SAR were dead while the still alive plants exhibited poor growth with sparse leaves and visible damage. Seedlings under pH 2.0 NAR had significant areas of necrotic, yellowing and wilting leaves. These results suggested that heavy acid rain caused significant injuries to *Q. glauca* and the effect of SAR on plants was more severe than NAR. This was consistent with the results of Chen et al., where they found that the decrease in maximum net photosynthetic rate and light use efficiency in *Schima superba* seedlings under SAR were greater than NAR stress, and the seedlings treated with SAR had higher O_2_^−^ production rate than the NAR treatments [3]. Chen et al. also discovered that *Liquidambar formosana* seedlings sprinkled with SAR possessed higher H_2_O_2_ content, which were formed during various metabolic reactions leading to oxidative stress compared to plants treated with NAR [3,74].

### 4.6. Limitations and Recommendations 

Acid rain can affect plant health by causing damage to leaf surface, then the cell structure and enzyme levels, resulting in leaf reflectance changes [5,75]. However, we did not obtain health status data of *Q. glauca* at a cellular level or a smaller scale in this study. Future developments in this field can use some novel machines and techniques to extract more detailed spectral information and health status data of plants. Additionally, the experimental time was much less than that of plants suffering acid rain in Nature, so natural acid rain could be more harmful to plants than simulated acid rain. In future studies, it is urgent to integrate remote sensing information with ground-based hyperspectral data, this would help to extract spectral information to monitor and diagnose acid rain stress on plants on a regional scale [19,28]. Radiative transfer model could be considered to upscale leaf-level spectral information ground data to airborne and satellite scales and negate the atmospheric noise effects [76,77].

## 5. Conclusions

In this study, a hyperspectral index (GPAI) is proposed to detect the long-term responses of *Q. glauca* to acid rain stresses. The results showed that acid rain could significantly influence the spectral information near the green peak (500–660 nm) in the visible region, and GPAI could track well the responses of *Q. glauca* under SAR and NAR treatments.

Under light SAR (pH 4.5) stress, GPAI difference relative to pH 5.6 SAR treatment increased with increasing experimental days, suggesting pH 4.5 SAR has a positive effect on *Q. glauca*; the moderate SAR (pH 3.0) changed the GPAI difference between pH 3.0 and pH 5.6 SAR treatments from positive to negative at about 300 days of acid rain treatment; GPAI difference between pH 2.0 and pH 5.6 SAR significantly decreased over time, and reached to negative at about 50 days of acid rain treatment, suggesting heavy SAR (pH 2.0) damaged to *Q. glauca* at the initial stage and the injury to seedlings enhanced with the treatment time increasing.

Compared to the NAR control group (pH 5.6), GPAI of *Q. glauca* under pH 4.5 NAR significantly increased with time, while the GPAI difference between pH 3.0 and pH 5.6 NAR was at a low level negative value throughout the experiment, suggesting pH 4.5 NAR had a strong promoting effect on *Q. glauca* growth and pH 3.0 NAR mildly inhibited the seedlings; similar to pH 2.0 SAR, GPAI difference under pH 2.0 NAR treatment decreased with increasing experimental days.

This study suggested that plant responses caused by acid rain stress could be distinguished by hyperspectral information. From the experimental results and the responses of GPAI, the influence of SAR on plants was more pronounced and severe than NAR. This is the first study to detect and compare the long-term responses of plant under SAR and NAR stresses using hyperspectral data, which provides a scientific basis for monitoring plants under acid rain stress at larger spatial scales using satellite data.

## Figures and Tables

**Table 1:** The average of rainfall and temperature (T) in Lin’an District from 1951 to 2008 (mm, °C).

		Winter			Spring			Summer			Autumn	
Month	12	1	2	3	4	5	6	7	8	9	10	11
Rainfall	51	72	85	125	127	157	211	147	148	150	78	61
Max T	11	8	10	14	21	25	29	33	33	28	23	17
Min T	3	1	3	6	12	17	21	25	25	21	15	9
